# A comparison of whole genome sequencing with exome sequencing for family-based association studies

**DOI:** 10.1186/1753-6561-8-S1-S38

**Published:** 2014-06-17

**Authors:** Sean Lacey, Jae Yoon Chung, Honghuang Lin

**Affiliations:** 1 Department of Biostatistics, Boston University School of Public Health, 801 Massachusetts Avenue 3rd Floor, Boston, MA 02118, USA; 2 Bioinformatics Program, Boston University, 44 Cummington Mall, Boston, MA 02215, USA; 3 Department of Medicine, Boston University School of Medicine, 72 East Concord Street, Boston, MA 02118, USA

## Abstract

As the cost of DNA sequencing decreases, association studies based on whole genome sequencing are now becoming feasible. It is still unclear, however, how much more we could gain from whole genome sequencing compared to exome sequencing, which has been widely used to study a variety of diseases. In this project, we performed a comparison between whole genome sequencing and exome sequencing for family-based association analysis using data from Genetic Analysis Workshop 18. Whole genome sequencing was able to identify several significant hits within intergenic regions. However, the increased cost of multiple testing counteracted the benefits and resulted in a higher false discovery rate. Our results suggest that exome sequencing is a cost-effective way to identify disease-related variants. With the decreasing sequencing cost and accumulating knowledge of the human genome, whole genome sequencing has the potential to identify important variants in regulatory regions typically inaccessible for exome sequencing.

## Background

Over the past few years, genome-wide association studies (GWAS) have successfully identified thousands of genetic loci associated with a variety of diseases and phenotype traits [1]. However, because of the limited resolution of microarray-based genotyping platforms, a vast majority of the human genome is not yet genotyped directly in GWAS. Since 2004, the advance of next-generation sequencing technologies has substantially lowered the cost of DNA sequencing. Nevertheless, it is still expensive to perform whole genome sequencing on a large cohort of samples, so reducing the cost by sequencing the most informative regions is a desirable approach. The human exome consists of 1% of the human genome but harbors 85% of disease-related variants [2]. Therefore, the cost of exome sequencing is typically only one-sixth that of whole genome sequencing [3]. Several commercial exome-capture platforms are currently available, each with a different design focus [4-6].

It is, however, still unclear whether exome sequencing is able to capture genetic variants associated with complex diseases. The objective of this project is to examine how much we could gain from exome sequencing compared with whole genome sequencing.

## Methods

For this study, we used a pedigree-based sample from the Type 2 Diabetes Genetic Exploration by Next-Generation Sequencing in Ethnic Samples (T2D-GENES) Consortium provided by the Genetics Analysis Workshop 18 (GAW18). Whole genome sequence data was available for 959 participants from 20 families; 464 participants were directly sequenced and 495 were imputed from GWAS data. This data was cleaned of Mendelian errors prior to distribution. A total of 8,348,674 single-nucleotide polymorphisms (SNPs) were identified across all the odd-numbered chromosomes. Among them, 4,152,114 were common variants with a minor allele frequency (MAF) ≥1%. In addition, 425,734 common variants were identified by GWAS.

The data set contains phenotypes measured at 4 exams. At each exam, the following characteristics were recorded: age, hypertension, systolic blood pressure (SBP), diastolic blood pressure (DBP), use of blood pressure medications, and smoking status. Hypertension is associated with a variety of diseases, such as stroke [7], diabetes [8], and heart failure [9]. The use of blood pressure medications would counteract the effect of genetic variations and introduce bias to the association analysis, so we excluded subjects using blood pressure medications or without covariate information.

This project used SBP at the baseline exam as the primary outcome, both for real and simulated phenotypes. The data was preprocessed using the PLINK software package [10]. Our model included SBP as the response variable and genotype data as the independent variable adjusting for age, gender, and smoking status. To account for family structure, we used a linear mixed-effects model as implemented in the "kinship" R package. The functional implication of genetic variations was predicted by ANNOVAR [11].

Because only the whole genome sequence was available, we mimicked the exome sequence by restricting the analysis to targeted regions designed by the 3 most common commercial exome capture platforms, Agilent SureSelect Human All Exon 50Mb, NimbleGen SeqCap EZ Exome Library v2.0, and Illumina TrueSeq Exome Enrichment. We assumed all the SNPs within the targeted regions were successfully captured. (Table 1 lists the number of SNPs captured by each exome platform.) On average, each platform captured approximately 133,000 SNPs, or approximately 1.6% of whole genome sequencing.

**Table 1:** Number of variants captured by each platform

Platform	Number of variants	Number of common variants
Whole genome sequencing	8,348,674	4,152,114
Exome sequencing (Agilent)	129,204	58,091
Exome sequencing (Illumina)	156,910	70,347
Exome sequencing (NimbleGen)	113,150	50,000
GWAS SNPs	453,285	425,734

## Results

**Figure 1:**
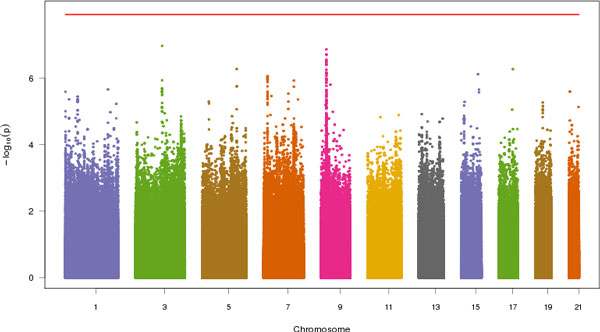
**Manhattan plot of common variants associated with SBP**. Red line is the Bonferroni *p *value cutoff.

Figure 1 shows the Manhattan plot of common variants across all odd-numbered chromosomes. A few peaks could be observed in chromosomes 3 and 9, indicating that the variants at these loci might be associated with SBP. Table 2 lists the top 30 SNPs from whole genome sequencing, and Table 3 lists the top 10 SNPs from GWAS together with 3 exome-sequencing platforms. The most significant SNP for the whole genome sequencing is chr3:106206487 (rs2590204, *p *= 1.1 × 10^−7^). The SNP is located within a gene desert, and the closest gene is *CBLB*, which encodes the E3 ubiquitin-protein ligase. The remaining top SNPs are all located within the introns or upstream of *PSIP1*, which is why they were not captured by the 3 exome platforms. The most significant SNP identified by all 3 exome platforms was chr7:11022564 (rs218965, *p *= 8.8 × 10^−7^), which is a synonymous mutation in *PHF14*. GWAS was able to pick up another SNP, chr9:15472139 (rs2777950, *p *= 7.5 × 10^−7^), which is located within the introns of *PSIP1*.

**Table 2:** Most significant variants in whole genome sequencing

Rank	SNP	*p *Value	Function	Rank	SNP	*p *Value	Function	Rank	SNP	*p *Value	Function
1	3:106206487	1	Intergenic	11	5:144654771	5.3E-07	Intergenic	21	7:11022564	8.8E-07	Exonic
2	9:15503905	1.3E-07	Intronic	12	17:63733954	5.3E-07	Intronic	22	7:11025635	8.8E-07	Intronic
3	9:15500315	1.9E-07	Intronic	13	9:15396745	5.5E-07	Intergenic	23	7:11027754	8.8E-07	Intronic
4	9:15501713	1.9E-07	Intronic	14	9:15527348	6.7E-07	Intergenic	24	7:11016614	9.4E-07	Intronic
5	9:15501753	1.9E-07	Intronic	15	9:15501422	7.1E-07	Intronic	25	7:11008221	1.0E-06	Intergenic
6	9:15500150	2.8E-07	Intronic	16	9:15472139	7.5E-07	Intronic	26	9:15459251	1.0E-06	Intronic
7	9:15528010	3.1E-07	Intergenic	17	15:92024157	7.5E-07	Intergenic	27	7:11022230	1.0E-06	Exonic
8	9:15482976	3.3E-07	Intronic	18	9:15396695	8.4E-07	Intergenic	28	7:11025638	1.1E-06	Intronic
9	9:15498495	3.7E-07	Intronic	19	7:11016690	8.8E-07	Intronic	29	7:11015444	1.1E-06	Intronic
10	9:15466033	4.0E-07	Intronic	20	7:11018224	8.8E-07	Intronic	30	9:15397233	1.1E-06	Intergenic

**Table 3:** Most significant variants in exome sequencing and GWAS

Rank	GWAS SNPs			Agilent			Illumina			NimbleGen		
	SNP	*p *Value	Function	SNP	*p *Value	Function	SNP	*p *Value	Function	SNP	*p *Value	Function
1	9:15472139	7.5E-07	Intronic	7:11022564	8.8E-07	Exonic	7:11022564	8.8E-07	Exonic	7:11022564	8.8E-07	Exonic
2	7:11022564	8.8E-07	Exonic	7:11022230	1.0E-06	Exonic	7:11022230	1.0E-06	Exonic	7:11022230	1.0E-06	Exonic
3	7:11008221	1.0E-06	Intergenic	9:15468480	4.4E-06	Intronic	7:94927677	2.9E-06	UTR3	9:15571630	3.2E-05	Exonic
4	7:11022230	1.0E-06	Exonic	7:41661724	2.9E-05	Intergenic	7:94921491	8.3E-06	UTR3	9:14863863	7.2E-05	Exonic
5	7:11015444	1.1E-06	Intronic	9:15571630	3.2E-05	Exonic	9:15571630	3.2E-05	Exonic	3:125859012	7.4E-05	Intronic
6	9:15528290	1.1E-06	Intergenic	3:184766392	3.9E-05	Intronic	3:184770380	3.9E-05	UTR3	17:64210580	7.5E-05	Exonic
7	9:15443430	1.4E-06	Intronic	3:184769911	4.5E-05	UTR3	3:184769911	4.5E-05	UTR3	7:44608718	7.9E-05	Intronic
8	9:15469733	1.7E-06	Intronic	3:184769941	4.5E-05	UTR3	3:184769941	4.5E-05	UTR3	1:212798260	1.0E-04	Exonic
9	3:106199956	2.2E-06	Intergenic	9:14863863	7.2E-05	Exonic	7:94921543	6.8E-05	UTR3	1:210761365	1.2E-04	Exonic
10	9:15446868	2.3E-06	Intronic	17:64210580	7.5E-05	Exonic	9:14863863	7.2E-05	Exonic	7:2414142	1.2E-04	Intronic

Because we performed thousands of tests, it is likely that many SNPs were false positives even if they reached the nominal significance cutoff. The simplest way to adjust for multiple testing is by Bonferroni correction [12], which uses a cutoff equivalent to 0.05 divided by the number of tests. So a SNP is claimed significant only if its *p *value is less than 1.2 × 10^−8 ^for the whole genome sequencing. Given that less than 2% of SNPs were tested in exome sequencing, the *p *value cutoffs would be 8.6 × 10^−7^, 7.1 × 10^−7^, and 6.1 × 10^−7 ^for Agilent, Illumina, and NimbleGen, respectively. For GWAS, the *p *value cutoff would be 1.2 × 10^−7^. Given these cutoffs, none of our top SNPs were significant.

**Figure 2:**
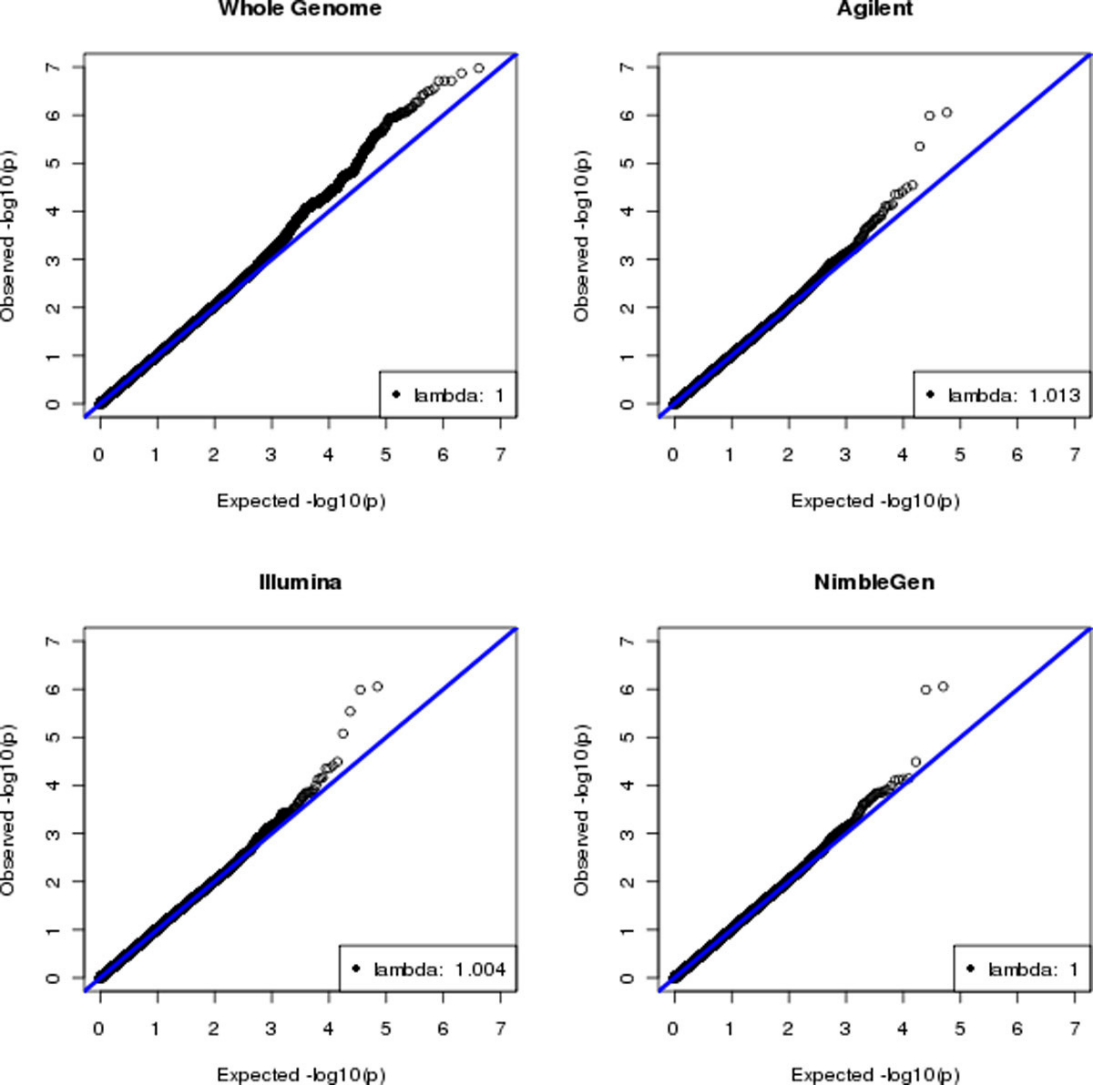
Q-Q plots of common variants associated with SBP for whole genome sequencing and exome sequencing

However, Bonferroni correction is usually too conservative because of the linkage disequilibrium between SNPs. Several studies have been conducted to estimate the appropriate significance cutoffs for genetic tests [13-15]. Here we chose the false discovery rate (FDR) [16] to control type I error. Table 4 shows the number of significant SNPs that met different FDR thresholds in each platform. With FDR <5%, all 3 exome platforms had 2 significant SNPs, whereas none of GWAS SNPs or whole genome SNPs were significant. With the increasing FDR, we observed that more SNPs became significant. The most significant SNP for whole genome sequencing reached 13% of FDR. The results can be visualized in the Q-Q plots in Figure 2. Two SNPs in exome sequencing obviously deviated from the diagonal line, suggesting that they were significantly associated with SBP. Such deviation is absent from the whole genome sequencing. No inflated type I error was observed in all the platforms because the genomic control λ was close to 1.

**Table 4:** Number of SNPs passing FDR threshold in each platform

**FDR threshold**	**Agilent**	**Illumina**	**NimbleGen**	**GWAS**	**Whole genome**
5%	2	2	2	0	0
10%	3	3	2	10	0
15%	3	4	2	18	40

We also performed association tests on the 200 simulated SBP phenotypes. The analysis was limited to SNPs at chromosome 3 because of the computational burden. On average, whole genome sequencing identified 163 significant hits per run, whereas exome sequencing identified 10, 7, and 7 for Agilent, Illumina, and NimbleGen, respectively. However, because over 50 times more SNPs were tested, whole genome sequencing did not show a significant advantage over exome sequencing in terms of identifying independent loci.

## Discussion

In this project, we compared the performance of whole genome sequencing with exome sequencing in a family-based association study. After correcting for multiple testing, we did not find great benefit from whole genome sequencing compared to exome sequencing. Our results suggest that exome sequencing is a cost-effective way to capture disease-related variants. Given the lower cost, exome sequencing allows a larger number of samples to be sequenced, which would significantly increase the statistical power for association studies.

One advantage of exome sequencing is that it focuses on the most informative proportion of the human genome. Therefore, the results are quite straightforward to interpret. It also lowers the requirement for computational resources and data storage. For example, the volume of exome sequence data is less than one-fifteenth that of whole genome sequence data. In addition, tools to efficiently analyze whole genome sequencing are still at an early stage.

Nevertheless, exome sequencing also has several intrinsic problems. Because of its heterogeneous capture capability, exome sequencing might introduce bias as a result of fragment size and GC content, which could result in ambiguous mapping and reduce the depth of coverage in the targeted regions. Exome sequencing also has very limited power to detect structural variations that are important to many diseases [17]. It is also worth noting that most of high penetrance variants that cause Mendelian diseases are very rare, thus association analysis is usually not the best way to study these diseases. Linkage analysis traditionally has been used for the purpose. Given that many common variations were located outside of the exome, whole genome sequencing would provide a better resolution for linkage analysis. We anticipate that the decreasing cost of sequencing and recent efforts to annotate the functional genome (eg, the ENCODE project [18]) will make whole genome sequencing more attractive and eventually lead to the retirement of exome sequencing.

## Conclusions

Exome sequencing is an effective method to identify disease-related variants in family-based association studies. As the cost of sequencing drops and our knowledge of the functional genome improves, we anticipate that whole genome sequencing will prove to be a better solution for future genetics research.

## Competing interests

The authors declare that they have no competing interests.

## Authors' contributions

HL conceived of study idea, guided analyses, and the edited manuscript. SL conducted statistical analyses and drafted the manuscript. JYC conducted statistical analyses and edited manuscript. All authors read and approved the final manuscript.
